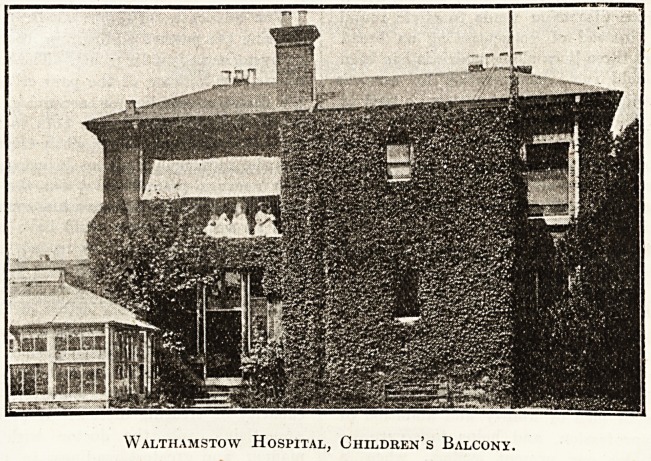# The Royal, the Humorous, the Heroic

**Published:** 1914-05-23

**Authors:** 


### SOME HISTORIC CHILDREN'S HOSPITAL COTS.

### The Royal, the Humorous, the Heroic.

Among named hospital cots are many which commemorate the long reign of Queen Victoria. One is to be found at Walthamstow, and another at the Royal Waterloo Hospital, both of which were endowed by public subscription; Gateshead has one endowed in 1897 in commemoration of the Diamond Jubilee, as well as one endowed in 1901—" The Victoria Memorial Cot."

Great Ormond Street possesses a cot, the "Alexandra," founded in perpetuity by Queen Alexandra with a sum presented to Her Majesty at the Imperial Coronation Bazaar by the members of the London Stock Exchange; as well as the "Mary Victoria," endowed in 1903 by the "Children's Salon" in connection with the *Gentlewoman*, for the lifetime of Princess Mary Victoria of Wales.

The history of royal visits is also writ upon the memorial tablets of many a cot. That at the Manchester Children's Hospital commemorates the visit of King Edward VII. and Queen Alexandra to Salford on July 13, 1905; and the "Prince of the Asturias" cot at the Belgrave tells of a hospital visit of Her Majesty the Queen of Spain. A cot which is a royal one and at the same time commemorates a hero's death is to be found at the Queen's Hospital for Children, Hackney Road; Prince Christian Victor died in 1900, during the Boer War, and the cot which bears his name was established *in memoriam* by the "Children's Salon."

It speaks volumes for what lies behind the British Navy when there is to be seen in a children's hospital a bed named and supported by a ship's company; but this is so at the Queen's Hospital for Children, for there the visitor will notice the "H.M.S. *Black Prince*" cot, which has now for several years been supported by that first-class cruiser.

It is also fitting that the memory of one who delighted in the society of little children should be perpetuated in an institution whose aim is the alleviation of the sufferings of the little ones; thus it was that the friends of the Rev. C. L. Dodgson, in a desire to erect a memorial, endowed in 1898 in the Hospital for sick Children the "Lewis Carroll" cot to the memory of the mathematical author of "Alice in Wonderland."

In January 1900 the very serious financial position of the Great Ormond Street Hospital was brought to the notice of "Mr. Punch," and an appeal was issued by him to his many readers in a touching story by Mr. Anstey Guthrie, entitled "Two Visits," in which the writer depicts "Mr. Punch" visiting a children's hospital. He goes after much persuading and with many misgivings, but the impression is the reverse of painful—flowers, toys, goldfish are in profusion, and "Mr. Punch" takes his leave with a promise to "come again soon." The promise is not forgotten, and one day "Mr. Punch" decides upon a second visit to the hospital; he goes, finds his way to the ward best known to him, but there is no sister, no nurse, no little patients! He goes to another ward, but it, too, is empty; he meets the matron, and asks if all are already cured. Alas! no, they have been sent home—the hospital is closed, there are no funds! "If I had only come before," he cries, "I might have prevented it! I would have appealed myself in my own paper to my own public not to let such a thing be!" And so he did. Mr. Guthrie's story and the appeal of Mr. Burnand so moved the readers of *Punch* that donations and subscriptions flowed in from all parts of the United Kingdom, Canada, South Africa, Australia, Russia, Germany, and Greece, so that not only was it possible to pay off the debt of £4,500 due to the bankers, but sufficient funds were provided for the immediate needs of the hospital, and the sum of £1,000 was applied by the committee for the endowment of the "Mr. Punch" cot in the Alice Ward.

Walthamstow Hospital, Children's Balcony.

Just as there is the cot which marks the crisis in an institution's life, so there is to be seen one which calls to mind a national one, and such is the "Lucy Frances Lucas" at Great Ormond Street, founded in 1900 by Mrs. Lionel Lucas in memory of those who in the hour of their nation's danger laid down their lives in the Boer War.

There is a touch of musical humour in the naming of a cot at the Jenny Lind Children's Hospital, Norwich, for in one of the wards of that institution founded to the memory of the great singer is to be found a bed supported by a minstrel troupe!

## Figures and Tables

**Figure f1:**